# Sudden Cardiac Death, Post-Mortem Investigation: A Proposing Panel of First Line and Second Line Genetic Tests

**DOI:** 10.3390/jpm14050544

**Published:** 2024-05-20

**Authors:** Fabio Del Duca, Alessandro Ghamlouch, Alice Chiara Manetti, Gabriele Napoletano, Elena Sonnini, Biancamaria Treves, Alessandra De Matteis, Raffaele La Russa, Mary N. Sheppard, Vittorio Fineschi, Aniello Maiese

**Affiliations:** 1 Department of Anatomical, Histological, Forensic and Orthopedic Sciences, Sapienza University of Rome, Viale Regina Elena 336, 00161 Rome, Italy; fabio.delduca@uniroma1.it (F.D.D.); alessandro.ghamlouch@uniroma1.it (A.G.); gabriele.napoletano@uniroma1.it (G.N.); biancamaria.treves@uniroma1.it (B.T.); alessandra.dematteis@uniroma1.it (A.D.M.); vittorio.fineschi@uniroma1.it (V.F.); 2 Department of Public Health and Infectious Diseases, Sapienza University of Rome, 00168 Rome, Italy; alicechiara.manetti@uniroma1.it; 3 Medicina Genomica, Dipartimento Scienze della Vita e Sanità Pubblica, Università Cattolica del Sacro Cuore, 00168 Rome, Italy; elena.sonnini01@icatt.it; 4 Department of Clinical Medicine, Public Health, Life Sciences, and Environmental Sciences, University of L’Aquila, 67100 L’Aquila, Italy; 5 Department of Cardiovascular Pathology, Level 1, Jenner Wing Corridor 4, St George’s University of London, Cranmer Terrace, London SW17 0RE, UK; msheppar@sgul.ac.uk; 6 Department of Surgical Pathology, Medical, Molecular and Critical Area, Institute of Legal Medicine, University of Pisa, 56126 Pisa, Italy

**Keywords:** sudden cardiac death, molecular investigations, genetic tests, genes, cardiomyopathy, channelopathy, post-mortem, autopsy, molecular autopsy

## Abstract

Investigating the causes of Sudden cardiac death (SCD) is always difficult; in fact, genetic cardiac conditions associated with SCD could be “silent” even during autopsy investigation. In these cases, it is important to exclude other aetiology and assist to ask for genetic investigations. Herein, the purpose of this review is to collect the most-implicated genes in SCD and generate a panel with indications for first line and second line investigations. A systematic review of genetic disorders that may cause SCD in the general population was carried out according to the Preferred Reporting Item for Systematic Review (PRISMA) standards. We subsequently listed the genes that may be tested in the case of sudden cardiac death when the autopsy results are negative or with no evidence of acquired cardiac conditions. To make genetic tests more specific and efficient, it is useful and demanded to corroborate autopsy findings with the molecular investigation as evident in the panel proposed. The genes for first line investigations are HCM, MYBPC3, MYH7, TNNT2, TNNI3, while in case of DCM, the most implicated genes are LMNA and TTN, and in second line for these CDM, ACTN2, TPM1, C1QPB could be investigated. In cases of ACM/ARVC, the molecular investigation includes DSP, DSG2, DSC2, RYR2, PKP2. The channelopathies are associated with the following genes: SCN5A, KCNQ1, KCNH2, KCNE1, RYR2. Our work underlines the importance of genetic tests in forensic medicine and clinical pathology; moreover, it could be helpful not only to assist the pathologists to reach a diagnosis, but also to prevent other cases of SCD in the family of the descendant and to standardise the type of analysis performed in similar cases worldwide.

## 1. Introduction

Sudden Death (SD) is a “rapid (without any specific chronologic limit) and unexpected or unforeseen—both subjectively and objectively—death which occurs without any clinical evaluation and in apparently healthy people in normal activity (primary or unexpected sudden death) or in patients in an apparently benign phase during the course of a disease (secondary or expected sudden death)” [1].

Sudden Cardiac Death (SCD) can be used when SD occurs as a consequence of any congenital or acquired cardiac condition, whether already known to be present during life or identified during the autopsy as the probable cause of death; in contrast, there are cases where no obvious extra-cardiac causes were identified in autopsy and thus an arrhythmic event is the likely cause [2].

In the US, the incidence of SCD is in the range of 180–250,000 per year, while an estimated global incidence is in the range of 4–5 million cases per year [3]. The median age in the US is between 66 and 68 (males are more involved). Sports-related sudden deaths are higher in elite athletes with an incidence of 1:8253 per year, according to the National Collegiate Athletic Association (NCAA) [4].

In the case of SCD, it is important to investigate family conditions associated with possible genetic heart disease, ask for an autopsy, and when a possible cardiac genetic condition is found, it is appropriate to ask for a post-mortem genetic test(molecular autopsy). Furthermore, it is central in the case of cardiomyopathy to exclude other aetiology, for example, hypertension, obesity, and diabetes for cardiac hypertrophy and alcohol abuse for dilated cardiomyopathy. Moreover, toxicological analysis is essential to consider the possibility of drug abuse, which is frequently associated with cardiac electrical and contraction function [5]. Once other conditions are excluded, investigating genetic cardiomyopathy disease is mandatory.( Include reference Sheppard et al Genetic cardiomyopathies Virchows Archives 2023) 

To make genetic testing easier and faster, it could be useful to have a panel of the most frequent genes that result in cardiac disease. Their interpretation should be combined with the autopsy results and the clinical conditions of the subjectIt has recently been shown that combining postmortem testing and family investigation can lead to a diagnostic yield of 40% [6]. This research assumes a very important role in preventing SCD in all family members and relatives who need a cardiologic follow-up.

Herein, we provide a systematic literature review to collect data about the frequency of the most implicated genes in cardiac disease to propose a panel of genes to be used in first-line genetic investigations, and to advance some other useful genes for further research (second-line).

## 2. Materials and Methods

**Figure 1:**
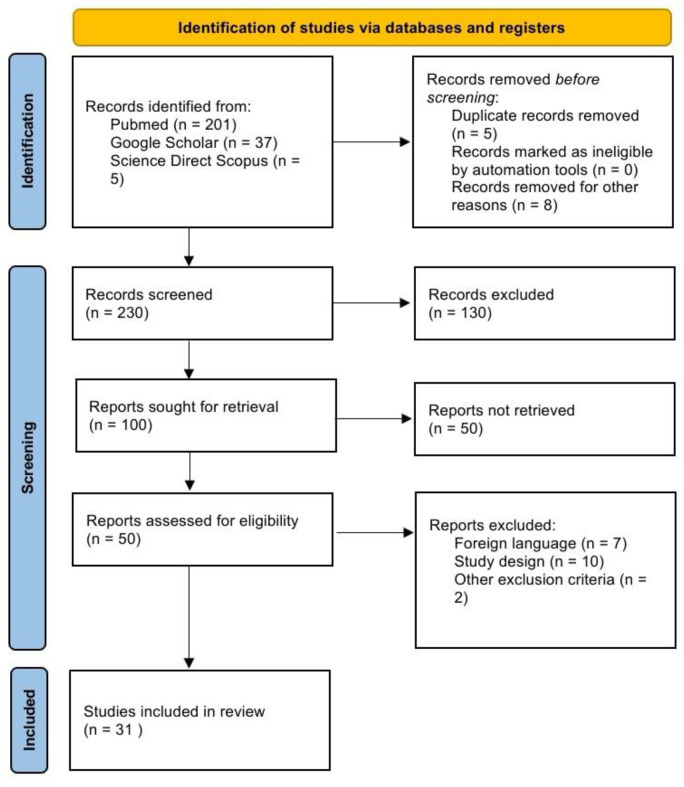
The selection of papers following the PRISMA protocol.

The present systematic review was carried out according to the Preferred Reporting Item for Systematic Review (PRISMA) standards. A methodological appraisal of each study was conducted according to the PRISMA standards, including an evolution of bias. PRISMA 2020 Statement was applied. It consists of a checklist and a flow diagram (Figure 1).

We performed a review of the English literature regarding genetic causes that can provoke SCD. A systematic literature search and critical review of the collected studies were conducted. An electronic search of PubMed (201), Google Scholar (37), and Science Direct Scopus (5) from database inception to March 2024 was performed. Databases were investigated using the following research terms (“post-mortem genetic test”) OR (“molecular autopsy results”) AND (“sudden cardiac death)”; in all fields [e.g., title, abstract, and keywords].

From this research, a list of abstracts was organized in the form of a dataset, and it was downloaded in a .nbib file and uploaded to Software Zotero 6-0.30, used as a citation manager.

The research group, following a meeting, established the inclusion and exclusion criteria for the paper, in accordance with PRISMA standards [7].

First, two investigators (A.G. and F.D.D.) read all the abstracts found in databases. The bibliographies of all identified papers were examined and cross-referenced to further identify relevant literature. After selecting abstracts and investigating the bibliographies of related papers, data collection began. One investigator (A.G.) independently examined papers with titles or abstracts that appeared to be relevant, selecting those that analysed the SCD due to genetic disease in children, adults, or young adult subjects.

The data collection process included study selection and data extraction. Disagreements concerning eligibility among the researchers were resolved by consensus. Only papers in English were included.

Data extraction was performed by two investigators (A.G. and F.D.D.) and verified by additional investigators (A.M., R.L.R., and ACM).

The current study provides a useful overview for those studying genetic conditions associated with sudden cardiac death. However, due to the huge number of genetic disorders and their ongoing discovery, it is important to continue documenting and sharing such cases while keeping the limitations of this type of research in mind.

## 3. Results

A review of the titles and abstracts, as well as a manual search of the reference lists, was achieved. The reference lists of all identified articles were reviewed to find missed literature. This search identified 100 papers, which were then screened based on their abstract. Among the resulting 100 articles, 50 were not retrieved, which left 50 articles for further consideration.

Papers in a non-English language were excluded [n. 7], and the following inclusion criteria were used: (1) original research articles, (2) reviews and mini-reviews, and (3) case reports/series. The mentioned publications were meticulously assessed, taking into account the primary objectives of the review. This evaluation left 31 scientific papers comprising original research articles, case reports, and case series.

The study sample, considering all the scientific papers analysed, was formed by 1050 cases of SCD. The included articles have been divided into two different groups, which are outlined in Table 1 and Table 2. In Table 1 we include the articles that report the main genes involved in different types of cardiomyopathy.

After reviewing the mutations in the genes mentioned in the studies, we subsequently listed those that may be tested in the case of sudden cardiac death when the autopsy results are negative or with no evidence of acquired cardiac conditions. We also found some genes associated with systemic genetic disorders that could contribute to SCD as Marfan disease or Fabry disease. To enhance comprehension, a list of coding genes is provided in Table 2.

### 3.1. Cardiomyophaty

Analysing all the articles, we found that the most frequent genes involved in HCM (hypertrophic cardiomyopathy) are MYBPC3 (included in 8 articles), MYH7 (included in 10 articles), TNNT2 (included in 7 articles), TNNI3 (included in 5 articles), while in DCM (dilated cardiomyopathy), they are LMNA (included in 8 articles), TTN (included in 8 articles), and in ACM (arrhythmogenic cardiomyopathy)/ARVC (Arrhythmogenic right ventricular cardiomyopathy), they are DSP (included in 10 articles), DSC2 (included in 5 articles), RYR2 (included in 9 articles), DSG2 (included in 4 articles), PKP2 (included in 5 articles).

It is important to underline that many of these genes could be implicated not only in one specific cardiomyopathy, but could interact and be mutated in all these cardiomyopathies.

Moreover, other genes are implicated in cardiomyopathy, but they are less frequent in the population, so they could be used as second-line molecular analysis when the principals’ genes are negative.

In fact, Van Driest et al. (2002) [8] in their study described a case of HCM associated with a rare mutation of TPM1, while Kraoua et al. (2022) [8] described a HCM associated with a mutation in the ACTN2 gene; another study by Webster et al. (2021) [23] shows an HCM associated with C1QPB mutation.

**Figure 2:**
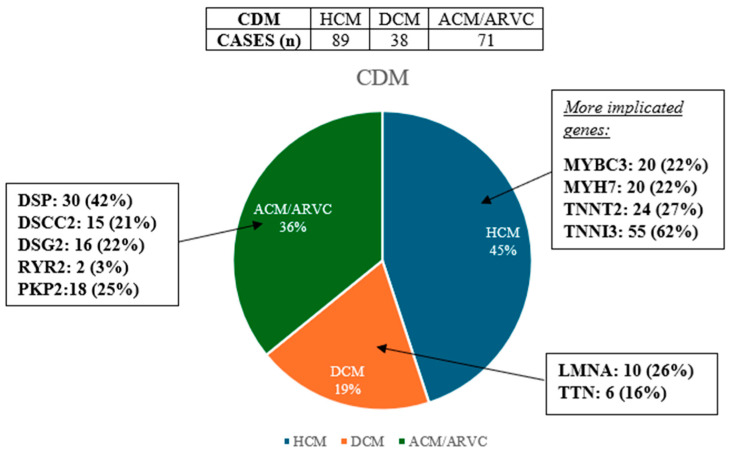
Percentage of CDM in the sample and main mutated genes.

In Figure 2, below we can see the percentage of the different CDM in the sample analysed.

### 3.2. Channelopathy and Arrhythmic Condition

In Table 3, we include the articles that report the main genes involved in different types of channelopathy and arrhythmic conditions that cause SCD. The most common genes are the ones which are implicated in the sodium and potassium channels: SCN5A (BrS), KCNQ1 (LQTS), KCNH2 (LQTS), KCNE1 (LQTS) for conditions like LQTS and BrS; additionally, for the CPVT, the most frequent gene is RYR2.

These genes tested positive in most of the articles about channelopathy and arrhythmic death. Indeed, SCN5A was positive in 15 articles, KCNQ1 in 10 articles, KCNH2 in 12 articles, KCNE1 in 6 articles, and RYR2 in 14 articles.

There were also sporadic conditions associated with arrhythmic death that could be found. Chugh et al. (2004) [30], among their SCD group, described two cases of Wolff-Parkinson-White (diagnosed on the 12-lead ECG); in their article, Neubauer et al. (2016) [14] described mutations in DCHS1 and TGFB2, related to Mitral valve prolapse (associated with mild systemic features of Marfan syndrome) that comport a SCD. Santori et al. (2015) [11] found a case of Fabry disease related to mutation of GLA.

## 4. Discussion

SCD could have many aetiologies. In fact, in younger people, most cases are due to congenital heart defects, while in over-35-year-old people, coronary heart diseases are the major cause. In 15% of the cases, SCD occurs in patients with non-ischaemic structural cardiomyopathies (as HCM, DCM, and ARVC), sarcoidosis, amyloidosis, and myocarditis. Moreover, valvular heart disease can also cause SCD (aortic stenosis with risk of 5–7% and mitral valve prolapse with a risk of 0.2–1.9%) due to two different conditions: mechanical and arrhythmogenic [41]. Likewise, performing laboratory test results is very important to exclude a systemic, life-threatening disorder like anaphylaxis [42].

The aim of this review is to show which genes could be useful to investigate in the case of SCD [43,44].

### 4.1. Hypertrophic Cardiomyopathy

HCM is one of the most common conditions that occurs in the heart. In the USA, Europe, Japan, China, and East Africa, it has a prevalence of at least two in 1000 in the general population [45]. Its clinical diagnosis requires a hypertrophied non-dilated left ventricle not associated with any other cardiac conditions or systemic disorder (e.g., history of hypertension, diabetes, or obesity). The mutations that cause HCM affect genes implicated in encoding thick and thin contractile myofilament protein components of the sarcomere or the adjacent Z- disc [46]. In our review, we found that the most implicated genes are MYBPC3, MYH7, TNNT2, and TNNI3, which are all involved in the formation of the basal unit of the muscle (sarcomere).

SCD in HCM is caused by an alteration in the cardiomyocyte architecture with fibrosis and increased deposition of the collagen matrix, increasing the probability of ventricular arrhythmias [47]. A reduction in diastolic filling of the small left cavity or the obliterative thickening of the arterioles (milking effect) leading to ischaemia may be other causes of SCD [1].

### 4.2. Dilated Cardiomyopathy

DCM is defined as the presence of left ventricular or biventricular dilatation or systolic dysfunction in the absence of abnormal loading conditions (e.g., primary valve disease, alcoholic/drug abuse, post-ischemic remodelling) [48]; the annual incidence is estimated at 5–8 cases per 100,000 people [49]. In this condition, SCD is due to electromechanical dissociation or ventricular arrhythmias and it occurs in up to 12% of patients, especially those with cardiac failure [50]. The most implicated genes are encoding for components of the sarcomere (LMNA and TTN) [51], 

### 4.3. Arrhythmogenic Cardiomyopathy

ACM is an inherited heart muscle disorder characterized by loss of myocytes and fibrofatty replacement of the right ventricular myocardium (ARVC), Lately it has been found that the pattern could also involve the left ventricular myocardium or both the ventricles [52]. The population prevalence of ACM has been estimated at 1:1000 to 5000 [53]. SCD may be the first manifestation of the condition, [54]. The genes implicated in ACM play a key role in the formation of desmosomal proteins. Our review shows these genes are frequently mutated in ACM (DSP, DSG2, DSC2, RYR2, PKP2).

### 4.4. Channelopathy

This disorder is caused by the alteration of the ion channels and includes conditions such as LQTS, Brugada Syndrome, SQTS, and CPVT. In these cases, autopsy findings are negative with a morphologically normal heart [1]. The prevalence of LQTS is estimated to be 1:2000 [55]; Brugada syndrome has a prevalence of 1:2000 [56].SQTS, this is a very rare condition with less than 300 cases described [57] CPVT has a prevalence of 1/10,000 in the general population [58]. D. The genes involved are associated with the alteration of ion channels most commonly SCN5A, KCNQ1, KCNH2, KCNE1, and RYR2.

Considering the SCD due to cardiomyopathy, our results highlight some frequent mutations and others that are sporadic. It could be useful to investigate the most frequent mutations with first-line molecular research, while the less frequent could be used as second-line research., we suggest the following genes as first-line molecular research: MYBPC3, MYH7, TNNT2, TNNI3 in the cases of genetic HCM; LMNA, TTN in the cases associated with DCM or DSP, DSG2, DSC2, RYR2, PKP2 for ACM. The second line may be in in ACTN2, TPM1, and C1QPB that resulted in mutation in 3 different sporadic cases of HCM and DCM. When the autopsy does not show any structuralcardiac anomaly, channelopathies he following panel of genes: SCN5A, KCNQ1, KCNH2, KCNE1, and RYR2 should be used.

A limit that a pathologist could face when approaching such cases is that it may be hard to get permission for genetic analysis, especially in peripheric centres; moreover, the communication of significant information to the family is difficult to standardize but it is essential to find a pathway that allows relatives to make informed decisions about whether or not they want to pursue both cardiological and genetic investigations [59].

**Figure 3:**
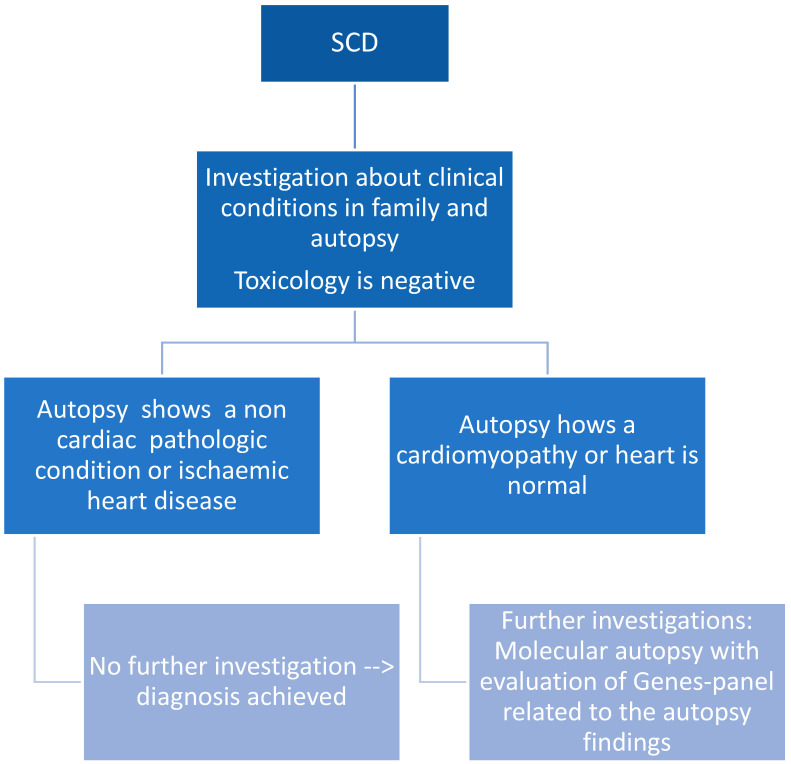
Algorithm of unexplained SCD.

**Figure 4:**
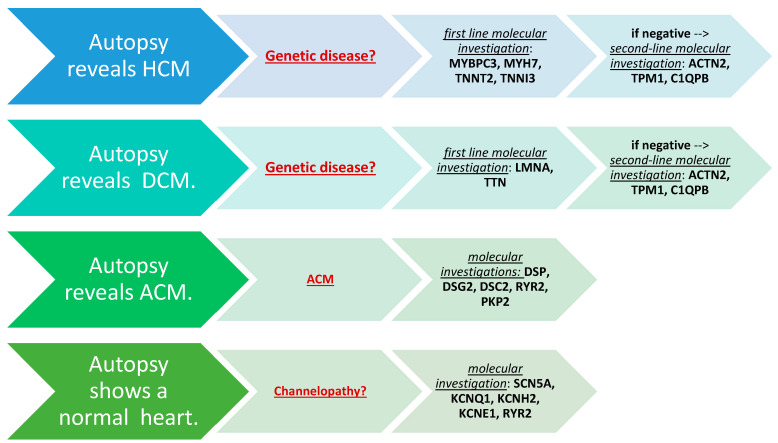
A panel of genes.

Below is a possible algorithm for SCD cases (Figure 3) and a panel of genes that may be investigated in SCD (Figure 4).

## 5. Conclusions

As our review highlighted, there is no standardised and internationally accepted algorithm regarding the appropriate genetic investigations in cases of SCD. This review underlines the importance of genetic tests in forensic medicine and clinical pathologyWe propose a panel of genes that should be studied as first- and second-line analysis in cases of SCD, according to the autopsyc findings. This will help the pathologists to correlate with their diagnosis, and also standardise the type of analysis performed in similar cases worldwide. Ccollecting comparable data, in different countries, is fundamental to gaining more knowledge about SCD worldwide, An accurate diagnosis of a cardiac death will save the lives of family members of the deceased, addressing them to a cardiological follow-up. The implementation of genetic tests and their use as a complementary analysis after the autopsy in cases of SCD represents the most relevant example of “mors gaudet succurrere vitae”.

## Figures and Tables

**Table 1:** Most-implicated genes in Cardiomyopathy.

References	n.	Age (Average)	Sex	Gene	Disease
Van Driest et al. (2002) [8]	1	8	F	TPM1	HCM
Di Gioia et al. (2006) [9]	100	2–40 (30.3)	M (69) F (31)	RYR2, KVLQT1, HERG, SCN5A	HCM, DCM, ARVC, Channelopathy
Larsen et al. (2011) [10]	41	0–40	M (/) F (/)	MYH7, MYBPC3, TNNI3, TNNT2, MYL2, MYL3, LMNA, PKP2, DSP, DSG2, DCS2, JUP, TMEM43	HCM, DCM, ARVC
Campuzano et al. (2014) [11]	29	21 days—14 (3.29)	M (14) F (15)	SCN5A, KCNQ1, KCNH2, KCNE1, KCNE2, KCNE3, RyR2, MYBPC3, MYH7, PKP2, DSC2, DSP, LMNA	ACM, HCM, DCM, LQTS, SQTS, BrS
Santori et al. (2015) [12]	41	18 weeks (SIDS:38) and 3 years (SADS:3)	M/F (74%, SIDS), M/F (67%, SADS)	MYBPC3, MYH6, JUP, LDB3, DSC2, TTN, MYLK2, AKAP9, FBN1, SCN5A, MYH7, RYR2, DSG2, DES, GLA, KCNE1L, MYL2, DSP, RANGRF, DMD, TNNT2, BAG3, SCN1B, RBM20	HCM, DCM, ACM, FABRY DISEASE, CHANNELOPATHY
Narula et al. (2015) [13]	14	1.3–29 (17.4)	M (8) F (6)	KCNQ1, KCNH2, SCN5A, RYR2, MYH7, MYBC3, TTN, CACNA1C, JPH2, VLC	HCM, DCM, ACM, Channelopathy
Hertz et al. (2015) [14]	72	1–50 (41)	M (50) F (22)	SCN5A, TRPM4, LDB3, LMNA, HCN4, DSP, MYBPC3, TTN, RYR2, NPPA, CACNA1C, MYH7, KCNA5, KCNQ1, KCNH2, CASQ2, MYH6, NEXN, AKAP9	HCM, ARCV
Neubauer et al. (2016) [15]	5	19–38 (29)	M (1) F (4)	DCHS1, TGFB2, GJD4, JPH2, DSP, KCNH2, MYH7, RANGRF, KCNQ1, SCN10A, SCN5A	MARFAN (MITRAL VALVE PROLAPSE), LQTS, ARVC, METABOLIC IMBALANCE
Hellenthal et al. (2017) [16]	10	19–40	M (5) F (5)	TTN, BAG3, DSG2, KCNH2, MYPN, CACNA1C, PRDM16, KCNE3, ABCC9, CACNB2, SCN5A, AKAP9, LAMA4, DSP, TNNT2	HCM, ARVC, DCM
Campuzano et al. (2017) [17]	52	14–50 (37.19)	M (48) F (4)	RYR2, TTN, ANK2, TNNT2, MYH7, PKP2, MYBPC3, DSC2, CACNA1C, NEXN, CACNB2, TNNI3, DSG2, CSRP3, HCN4, LAMP2 SGCD, CAV3, KCNH2, DSP, ANK2, MYH6, BAG3, HCN4, RBM20	ACM
Andersen et al. (2019) [18]	13	0 (SUDS) 29–50 (44, SADS)	M (10) F (3)	MYL2, MYL7, MYL4, PLN, FABP3, MYL3, TNNT2, ACTC1, TNNI3, MYH7, TTN, TNNC1, MB, DES, CKM, ACTA1, ANKRD1	DCM, HCM
Marey et al. (2020) [19]	35	21 days—72 (25.5)	M (18) F (17)	DSP, LMNA, TNNT2, TNNI3, MYBPC3, TTR	ARVC, HCM, DCM, RCM, LVNC
Grassi et al. (2020) [20]	1	7	F (1)	MYBPC3, MYH7, TNNI3, TNNT2, BAG3, LMNA, TTN, DSC2, DSP, PKP2, DSG2	HCM, DCM, ACM
Fernlund et al. (2020) [21]	10	0.1–24 (12.5)	M (8) F (2)	MYH7, ABCC9, FLNC, MYBPC3, PGM1, RBM20, ALPK3	HCM
Fahed et al. (2020) [22]	57	7–64	M (29) F (28)	TNNI3	HCM
Webster et al. (2021) [23]	2	7 months and 1 month	M (2)	C1QPB	HCM
Sen et al. (2021) [24]	1	42	M (1)	LMNA	DCM
Leone et al. (2021) [25]	1	13	M (1)	PKP2	ACM
Siskind et al. (2022) [26]	15	2 days—57 (15)	M (/) F (/)	RYR2, PRDM16, SCN10A, MYH7, MYBPC3, KCNH2, CASQ2, TRDN, SCN5A, MYH6, KCNA5, CACNA1C, MYLK2	CPVT, LQTS, ARVC, DCM, LVNC, BrS, HCM
Neubauer et al. (2022) [27]	10	6–55 (33.1)	M (9) F (5)	AKAP9, FHOD3, RBM20, LMNA, DSP, APOB, ABCC9, CDH2, JUP, MYBC3, RYR2, SCN10A, ZIC3, UBR4, ABCC6, GAA, MYOM1, FLNC, LDB3, LRRC10, MYH7, MYH11, SLC4A3, ACADM, LDB3, SLC22A5, AGPAT2, DSC2, SCN10A, TTN	ACM, DCM, SQTS, LQTS
Kraoua et al. (2022) [28]	2	28—3 and 10 months	M (2)	ACTN2	HCM
Votýpka et al. (2023) [29]	100	(33.3)	M (71) F (29)	GLA, KCNQ1, MYBPC3, SCN5A, RBM20, FHL1, TTN, FLNC, MYPN, COL3A1, TGFBR1, KCNH2, RYR2, TNNT2, DES, DSP, CTNNA3, PRKAG2, DPP6, LMNA, KCNE1	HCM, DCM, ARVC, LQTS

**Abbreviations:** HCM (hypertrophic cardiomyopathy), DCM (dilated cardiomyopathy), ACM (arrhythmogenic cardiomyopathy), ARVC (Arrhythmogenic right ventricular cardiomyopathy), LQTS (long QT Syndrome), SQTS (short QT Syndrome), BrS (Brugada Syndrome) and RCM (restrictive cardiomyopathy).

**Table 2:** Genes and their functions [30,31].

Genes	Functions	Sarcomeric or Not
LMNA	Provides instructions for making several slightly different proteins called lamins. The two major proteins produced from this gene, lamin A and lamin C, are made in most of the body’s cells. Lamins A and C are supporting (scaffolding) components of the nuclear envelope, which is a structure that surrounds the nucleus in cells.	Not sarcomeric
C1QPB	Is believed to be a multifunctional and multicompartmental protein involved in inflammation and infection processes, ribosome biogenesis, protein synthesis in mitochondria, regulation of apoptosis, transcriptional regulation, and pre-mRNA splicing.	Not sarcomeric
RYR2	Provides instructions for making a protein called ryanodine receptor 2. This protein is part of a family of ryanodine receptors, which form channels that transport positively charged calcium atoms (calcium ions) within cells.	Not sarcomeric
PKP2	Provides instructions for making a protein called plakophilin 2. This protein is found primarily in cells of the myocardium, which is the muscular wall of the heart.	Not sarcomeric
SCN5A	Belongs to a family of genes that provide instructions for making sodium channels. These channels open and close at specific times to control the flow of positively charged sodium atoms (sodium ions) into cells.	Not sarcomeric
KCNQ1	Belongs to a large family of genes that provide instructions for making potassium channels. These channels, which transport positively charged atoms (ions) of potassium out of cells, play key roles in a cell’s ability to generate and transmit electrical signals.	Not sarcomeric
KCNH2	Belongs to a large family of genes that provide instructions for making potassium channels. These channels, which transport positively charged atoms (ions) of potassium out of cells, play key roles in a cell’s ability to generate and transmit electrical signals.	Not sarcomeric
KCNE1	Belongs to a large family of genes that provide instructions for making potassium channels. These channels, which transport positively charged atoms (ions) of potassium out of cells, play key roles in a cell’s ability to generate and transmit electrical signals.	Not sarcomeric
MYBPC3	Delivers instructions for making cardiac myosin binding protein C (cardiac MyBP-C).	Sarcomeric
MYH7	Provides instructions for making a protein known as the beta (b)myosin heavy chain (sarcomere).	Sarcomeric
TNNT2	Provides instructions for making a protein called cardiac troponin T, which is found solely in the heart (cardiac) muscle.	Sarcomeric
TNNI3	Provides instructions for making a protein called cardiac troponin I, which is found solely in the heart (cardiac) muscle.	Sarcomeric
TTN	Provides instructions for making a very large protein called titin. This protein plays an important role in skeletal muscles, which the body uses for movement, and in heart (cardiac) muscle.	Sarcomeric
ACTN2	Encodes alpha-actinin-2, a protein expressed in human cardiac and skeletal muscle. The protein, located in the sarcomere Z-disk, functions as a link between the anti-parallel actin filaments.	Sarcomeric
TPM1	Essential sarcomeric component, stabilising the thin filament and facilitating actin’s interaction with myosin.	Sarcomeric
DSP	Provides instructions for making a protein called desmoplakin. This protein is found primarily in cells of the heart and skin, where it is a major component of specialised structures called desmosomes.	Sarcomeric
DSG2	Encodes a member of the desmoglein family and cadherin cell adhesion molecule superfamily of proteins. Desmogleins are calcium-binding transmembrane glycoprotein components of desmosomes, cell–cell junctions between epithelial, myocardial, and other cell types.	Sarcomeric
DSC2	Provides instructions for making a protein called desmocollin-2. This protein is found in many tissues, although it appears to be particularly important in the heart muscle and skin. Desmocollin-2 is a major component of specialized structures called desmosomes.	Sarcomeric

**Table 3:** Genes implicated in Channelopathy and other Arrhythmic SCD.

References	n.	Age (Average)	Sex	Gene	Disease
Chugh et al. (2004) [32]	12	-	-	KCNQ1 (KVLQT1), KCNH2 (HERG), SCN5A, KCNE1, and KCNE2	LQTS, WPW, BrS, CPVT
Di Gioia et al. (2006) [8]	100	2–40 (30.3)	M (69) F (31)	RYR2, KVLQT1, HERG, SCN5A	HCM, DCM, ARVC, CAD, Channelopathy
Campuzano et al. (2014) [10]	29	21 days—14 (3.29)	M (14) F (15)	SCN5A, KCNQ1, KCNH2, KCNE1, KCNE2, KCNE3, RyR2	ACM, HCM, DCM, LQTS, SQTS, BrS
Santori et al. (2015) [11]	41	18 weeks (SIDS:38) and 3 years (SADS:3)	M/F (74%, SIDS), M/F (67%, SADS)	MYBPC3, MYH6, JUP, LDB3, DSC2, TTN, MYLK2, AKAP9, FBN1, SCN5A, MYH7, RYR2, DSG2, DES, GLA, KCNE1L, MYL2, DSP, RANGRF, DMD, TNNT2, BAG3, SCN1B, RBM20	HCM, DCM, ACM, FABRY DISEASE, channelopathy
Stattin et al. (2015) [33]	15	(15)	M (10) F (5)	KCNQ1, KCNH2, RYR2, KCNE1, SCN5A	LQTS, CPVT, BrS, SQTS
Hertz et al. (2015) [13]	72	1–50 (41)	M (50) F (22)	SCN5A, TRPM4, LDB3, LMNA, HCN4, DSP, MYBPC3, TTN, RYR2, NPPA, CACNA1C, MYH7, KCNA5, KCNQ1, KCNH2, CASQ2, MYH6, NEXN, AKAP9	HCM, DCM, ARCV, LQTS, BrS
Narula et al. (2015) [12]	14	1.3–29 (17.4)	M (8) F (6)	KCNQ1, KCNH2, SCN5A, RYR2, MYH7, MYBC3, TTN, CACNA1C, JPH2, VLC	HCM, DCM, ACM, Channelopathy
Farrugia et al. (2015) [34]	16	2m—34	M (8) F (8)	KCNH2, SCN5A, ANK2, RYR2, KCNE1, CASQ2	LQTS, BrS, CPVT
Neubauer et al. (2016) [14]	5	19–38 (29)	M (1) F (4)	DCHS1, TGFB2, GJD4, JPH2, DSP, KCNH2, MYH7, RANGRF, KCNQ1, SCN10A, SCN5A	MARFAN (MITRAL VALVE PROLAPSE, LQTS, ARVC, METABOLIC IMBALANCE
Hellenthal et al. (2017) [15]	10	19–40	M (5) F (5)	TTN, BAG3, DSG2, KCNH2, MYPN, CACNA1C, PRDM16, KCNE3, ABCC9, CACNB2, SCN5A, AKAP9, LAMA4, DSP, TNNT2	HCM, ARVC, DCM, LQTS
Campuzano et al. (2017) [16]	52	14–50 (37.19)	M (48) F (4)	RYR2, TTN, ANK2, TNNT2, MYH7, PKP2, MYBPC3, DSC2, CACNA1C, NEXN, CACNB2, TNNI3, DSG2, CSRP3, HCN4, LAMP2, SGCD, CAV3, KCNH2, DSP, ANK2, MYH6, BAG3, PKP2, HCN4, RBM20	ACM
Lahrouchi et al. (2017) [35]	302	(24)	M (196) F (106)	KCNQ1, KCNH2, SCN5A, RYR2	CPVT, LQTS
Neubauer et al. (2018) [36]	34	1–63	M (26) F (8)	RYR2, ACAD9, AKAP9, SEMA3A	CPVT, BrS, LQTS
Neubauer et al. (2019) [37]	1	19	F (1)	SCN5A	LQTS
Mahlke et al. (2019) [38]	1	13	F (1)	RYR2	CPVT
Simons et al. (2021) [39]	1	49	M (1)	KCNQ1, DSG2	LQTS
Neubauer et al. (2022) [26]	10	6–55 (33.1)	M (9) F (5)	AKAP9, FHOD3, RBM20, LMNA, DSP, APOB, ABCC9, CDH2, JUP, MYBC3, SCN10A, ZIC3, UBR4, ABCC6, GAA, MYOM1, FLNC, LDB3, LRRC10, MYH7, MYH11, RYR2, SLC4A3, ACADM, LDB3, SLC22A5, AGPAT2, DSC2, SCN10A, TTN	ACM, DCM, SQTS, LQTS
Siskind et al. (2022) [25]	15	2 days—57 (15)	M (/) F (/)	RYR2, PRDM16, SCN10A, MYH7, MYBPC3, KCNH2, CASQ2, TRDN, SCN5A, MYH6, KCNA5, CACNA1C, MYLK2	CPVT, LQTS, ARVC, DCM, LVNC, BrS, HCM
Scheiper-Welling et al. (2022) [40]	56	1–50	M (/) F (/)	KCNH2, SCN5A, KCNQ1, MYBC3, VCL, JUP	ARRHYTMIC DEATH
Votýpka et al. (2023) [28]	100	(33.3)	M (71) F (29)	GLA, KCNQ1, MYBPC3, SCN5A, RBM20, FHL1, TTN, FLNC, MYPN, COL3A1, TGFBR1, KCNH2, RYR2, TNNT2, DES, DSP, CTNNA3, PRKAG2, DPP6, LMNA, KCNE1	HCM, DCM, ARVC, LQTS

**Abbreviations:** HCM (hypertrophic cardiomyopathy), DCM (dilated cardiomyopathy), ACM (arrhythmogenic cardiomyopathy), ARVC (Arrhythmogenic right ventricular cardiomyopathy), LQTS (long QT Syndrome), SQTS (short QT Syndrome), BrS (Brugada Syndrome), and RCM (restrictive cardiomyopathy); CPVT (Catecholaminergic Polymorphic Ventricular Tachycardia).

## Data Availability

Not applicable.

## Abbreviations

SCD (sudden cardiac death), HCM (hypertrophic cardiomyopathy), DCM (dilated cardiomyopathy), ACM (arrhythmogenic cardiomyopathy), ARVC (Arrhythmogenic right ventricular cardiomyopathy), LQTS (long QT Syndrome), SQTS (short QT Syndrome), BrS (Brugada Syndrome) and RCM (restrictive cardiomyopathy).
